# Association Between Findings on Videofluoroscopic Swallowing Studies and Pneumonia Onset in Residents of Elderly Care Facilities

**DOI:** 10.7759/cureus.105753

**Published:** 2026-03-24

**Authors:** Takafumi Yamano, Ryusei Nuita, Kensuke Kawamoto, Fumitaka Omori, Kaori Wada, Miho Tanaka

**Affiliations:** 1 Section of Otorhinolaryngology, Department of Medicine, Fukuoka Dental College, Fukuoka, JPN; 2 Department of Otorhinolaryngology, Fukuoka Dental College Hospital, Fukuoka, JPN; 3 Department of Nursing, Fukuoka Dental College Hospital, Fukuoka, JPN

**Keywords:** elderly care home, japanese geriatrics, pneumonia, swallow, videofluoroscopic swallowing studies

## Abstract

Introduction

Residents of elderly care facilities are at high risk of aspiration and frequently develop pneumonia. Despite this, swallowing function is often not assessed when pneumonia occurs. We evaluated the association between findings on videofluoroscopic swallowing studies (VFSSs) and the onset of pneumonia among elderly care home residents.

Methods

This retrospective study included residents admitted to a university-affiliated hospital for VFSSs following an acute illness. Participants were categorized into a pneumonia group (n = 29) and a non‑pneumonia group (n = 27). Six VFSS parameters (propulsion impairment, early pharyngeal entry, delayed laryngeal elevation time (LEDT), epiglottic vallecula residue, pyriform sinus residue, and laryngeal penetration or aspiration) were evaluated using established scoring criteria. Comparisons between groups were performed using the Mann-Whitney U test.

Results

Laryngeal penetration and aspiration were significantly more frequent in the pneumonia group (P = 0.015). No significant differences were observed between groups for propulsion impairment, early pharyngeal entry, LEDT prolongation, vallecular residue, or pyriform sinus residue. Composite scores for the oral and pharyngeal phases did not differ significantly; however, higher severity tended to occur in the pneumonia group.

Conclusion

Among the VFSS findings, laryngeal penetration or aspiration was significantly associated with pneumonia onset in residents of elderly care facilities. Considering the multifactorial nature of pneumonia development, swallowing assessment should be integrated with evaluation of oral hygiene, systemic health, cognitive function, and the care environment.

## Introduction

Elderly residents in care facilities are at increased risk of aspiration and, consequently, of pneumonia, including aspiration pneumonia, due to age‑related physiological decline, multimorbidity, diminished functional reserve, and medication effects such as sedatives and polypharmacy [1,2]. Aspiration is central to the pathophysiology of pneumonia among older adults, and residents of care facilities bear a greater pneumonia burden than community‑dwelling peers [1-3].

Following the onset of pneumonia, residents are typically transferred to acute‑care hospitals and receive antimicrobial and supportive therapy. However, despite the prominent role of dysphagia in aspiration pneumonia, instrumental swallowing assessments are not always performed before discharge. Contemporary reviews addressing pneumonia management in aging societies emphasize the need to systematize dysphagia (aspiration) assessment and embed it into clinical care pathways [3].

Videofluoroscopic swallowing studies (VFSSs) are core instrumental evaluations that dynamically visualize oral and pharyngeal swallowing physiology and yield key physiological markers that directly inform clinical decision‑making. These include the presence and severity of airway penetration or aspiration, quantified by the Penetration-Aspiration Scale (PAS); timing measures such as pharyngeal delay time (PDT) and pharyngeal transit time (PTT); and the degree and distribution of pharyngeal residue. In elderly patients with dysphagia, higher PAS scores, prolonged PDT/PTT, and increased vallecular or pyriform sinus residue have been associated with a history of pneumonia and with subsequent pneumonia events [4].

Fukuoka Dental University Medical and Dental Hospital operates an affiliated care facility, and many residents return there after recovering from pneumonia. Therefore, whenever feasible during hospitalization, the institution performs VFSSs to determine the feasibility of oral intake and to select appropriate diet textures [5].

Study objective

This study compares elderly care‑facility residents hospitalized for pneumonia with those admitted for non‑pneumonia reasons and identifies VFSS findings characteristic of pneumonia cases as well as their relationship to pneumonia onset. Clarifying these physiological features is expected to provide clinically actionable evidence to optimize swallowing rehabilitation, diet‑texture modification, and decisions regarding the continuation or restriction of oral intake for elderly residents returning to care facilities.

## Materials and methods

Facility background

The study facilities comprised one long-term-care health facility and two special nursing homes for older adults, all affiliated with a dental university and distinguished by comprehensive oral care protocols. Residents received professional oral care from dental hygienists after each meal, supplemented by weekly dental examinations conducted by dentists.

In Japan, care needs are determined through a nationally standardized certification system [6]. Trained staff from the local authority first visit the individual’s home or place of hospitalization and complete a multiple-choice questionnaire of approximately 90 items to assess physical and cognitive function, as well as utilization of medical services. Concurrently, the attending physician reports medical findings using a standardized form that combines multiple-choice responses with free-text descriptions.

Based on this information, individuals are provisionally classified into one of seven levels: Support Level 1 or 2, or Care Level 1-5, according to the estimated daily care time required [7]; higher levels indicate greater support and care needs. The residents included in our study all corresponded to Care Level 3 or higher, reflecting severe dependency, such as the inability to stand or walk independently and requiring full assistance with toileting, bathing, and dressing.

Subjects

Our study included 56 patients (12 males, 44 females) who developed an acute illness while residing in affiliated elderly care facilities and were admitted to our hospital for VFSSs. Patients were aged 70-108 years, with a mean age of 89.9 years. Patients with severe dementia, for whom compliance with instructions was difficult, were excluded. All participants were receiving oral intake during their facility stay; individuals receiving nasogastric tube feeding or with a gastrostomy were not included.

Evaluation method

Patients hospitalized with a diagnosis of pneumonia were assigned to the pneumonia group, while all others comprised the non-pneumonia group. Videofluoroscopic findings were compared between these groups.

Each parameter was assessed using a scoring system based on previously reported criteria [8]. Examinations were conducted with the patient in either a sitting or semi-sitting position. Omnipaque 300® (10 mL) was used as the contrast agent, and each examination was performed once. Six parameters were evaluated, including feeding disturbance, early pharyngeal influx, prolonged delay time of laryngeal elevation (LEDT), epiglottic vallecular retention, pyriform sinus retention, and laryngeal intrusion or aspiration. Each parameter was compared between the pneumonia and non-pneumonia groups.

Feeding disturbance was graded on a three-point scale as follows: 0 points indicated complete delivery of the contrast medium in a single swallow, 1 point indicated delivery of < 50%, and 2 points indicated delivery of ≥ 50%. Early pharyngeal entry was assessed on a two-point scale: 0 points indicated absence, and 1 point indicated presence. LEDT was used as a visual indicator of swallowing initiation during the pharyngeal phase. The normal value was defined as ≤ 0.35 seconds [9]. Prolongation was evaluated on a two-point scale: 0 points indicated absence, and 1 point indicated presence. Residual fluid in the epiglottic vallecula was graded on a four-point scale: 0 points indicated none, 1 point indicated a very small amount, 2 points indicated residue not overflowing from the epiglottic vallecula, and 3 points indicated overflow into the pharynx. Residual fluid in the pyriform sinus was graded similarly: 0 points indicated none, 1 point indicated a very small amount, 2 points indicated residue not overflowing into the larynx, and 3 points indicated overflow into the larynx. Laryngeal penetration and aspiration were graded on a three-point scale as follows: 0 points indicated no penetration or aspiration, 1 point indicated laryngeal penetration without aspiration, and 2 points indicated aspiration.

Statistical analyses were performed using IBM SPSS Statistics for Windows, Version 29 (Released 2023; IBM Corp., Armonk, New York). The Fukuoka Gakuen Research Ethics Committee approved the study protocol (approval no. 314).

## Results

Participant characteristics

Our study included 56 participants: 29 in the pneumonia group and 27 in the non-pneumonia group. The non-pneumonia group included participants aged 70-108 years, with a mean age of 90 years. The pneumonia group included participants aged 78-107 years, with a mean age of 90 years. No significant differences in mean age were observed between the groups. Sex distribution was as follows: the non-pneumonia group comprised 4 males and 23 females, whereas the pneumonia group comprised 8 males and 21 females. The proportion of male participants was higher in the pneumonia group compared to the non-pneumonia group.

Admission diagnoses in the non-pneumonia group

The most common diagnosis was urinary tract infection (UTI), occurring in 12 cases (44.4%). This was followed by femoral fracture in 3 (11.1%), cerebral infarction in 2 (7.4%), and gastrointestinal hemorrhage in 2 (7.4%). Other diagnoses included infectious enteritis, ischemic enteritis, liver abscess, acute cholecystitis, congestive heart failure, dehydration, epilepsy, and disuse syndrome, each accounting for 1 (3.7%) (Table 1).

**Table 1:** Admission Diagnoses in the Non-Pneumonia Group Distribution of primary diagnoses in the study cohort. Values are presented as n (%).

Diagnosis	n (%)
Urinary tract infection (UTI)	12 (44.4%)
Femoral fracture	3 (11.1%)
Cerebral infarction	2 (7.4%)
Gastrointestinal hemorrhage	2 (7.4%)
Infectious enteritis	1 (3.7%)
Ischemic enteritis	1 (3.7%)
Liver abscess	1 (3.7%)
Acute cholecystitis	1 (3.7%)
Congestive heart failure (CHF)	1 (3.7%)
Dehydration	1 (3.7%)
Epilepsy	1 (3.7%)
Disuse syndrome	1 (3.7%)

Comparison of VFSS findings

Feeding Disturbance

In the non-pneumonia group, 15 (55.6%) scored 0 points, 11 (40.7%) scored 1 point, and 1 (3.7%) scored 2 points. In the pneumonia group, 13 (44.8%) scored 0 points, 8 (27.6%) scored 1 point, and 8 (27.6%) scored 2 points. No significant difference was observed between the two groups (Table 2).

**Table 2:** Comparison of VFSS Findings (Non-Pneumonia Group vs. Pneumonia Group) - Feeding Disturbance No significant difference was observed between the two groups. VFSS: videofluoroscopic swallowing study

Score	Non-pneumonia Group	Pneumonia Group	Test Statistic, p-value
0	15 (55.6%)	13 (44.8%)	Mann–Whitney U = 309.5; z = −1.474; p = 0.140 (two‑sided)
1	11 (40.7%)	8 (27.6%)	
2	1 (3.7%)	8 (27.6%)	

Early Pharyngeal Entry

In the non-pneumonia group, 16 (59.3%) scored 0 points, and 11 (40.7%) scored 1 point. In the pneumonia group, 14 (48.3%) scored 0 points, and 15 (51.7%) scored 1 point. No significant difference was observed between the two groups (Table 3).

**Table 3:** Comparison of VFSS Findings (Non-Pneumonia Group vs. Pneumonia Group) - Early Pharyngeal Influx No significant difference was observed between the two groups. VFSS: videofluoroscopic swallowing study

Score	Non-pneumonia Group	Pneumonia Group	Test Statistic, p-value
0	16 (59.3%)	14 (48.3%)	Mann–Whitney U = 362.0; z = −0.562; p = 0.574 (two‑sided)
1	11 (40.7%)	15 (51.7%)	

LEDT Prolongation

In the non-pneumonia group, 14 (51.9%) scored 0 points, and 13 (48.1%) scored 1 point. In the pneumonia group, 11 (37.9%) scored 0 points and 18 (62.1%) scored 1 point. No significant difference was observed between the two groups (Table 4).

**Table 4:** Comparison of VFSS Findings (Non-Pneumonia Group vs. Pneumonia Group) - LEDT Prolongation No significant difference was observed between the two groups. VFSS: videofluoroscopic swallowing study

Score	Non-pneumonia Group	Pneumonia Group	Test Statistic, p-value
0	14 (51.9%)	11 (37.9%)	Mann–Whitney U = 344.0; z = −0.893; p = 0.372 (two‑sided)
1	13 (48.1%)	18 (62.1%)	

Residual Epiglottic Valley

In the non-pneumonia group, 15 (55.6%) scored 0 points, 4 (14.8%) scored 1 point, 8 (29.6%) scored 2 points, and 0 (0%) scored 3 points. In the pneumonia group, 13 (44.8%) scored 0 points, 9 (31.0%) scored 1 point, 5 (17.2%) scored 2 points, and 2 (6.9%) scored 3 points. No significant difference was observed between the two groups (Table 5).

**Table 5:** Comparison of VFSS Findings (Non-Pneumonia Group vs. Pneumonia Group) - Epiglottic Valley Residual No significant difference was observed between the two groups. VFSS: videofluoroscopic swallowing study

Score	Non-pneumonia Group	Pneumonia Group	Test Statistic, p-value
0	15 (55.6%)	13 (44.8%)	Mann–Whitney U = 363.5; z = −0.498; p = 0.619 (two‑sided)
1	4 (14.8%)	9 (31.0%)	
2	8 (29.6%)	5 (17.2%)	
3	0 (0%)	2 (6.9%)	

Persistence of the Pyriform Sinus

In the non-pneumonia group, 20 (74.1%) scored 0 points, 2 (7.4%) scored 1 point, 4 (14.8%) scored 2 points, and 1 (3.7%) scored 3 points. In the pneumonia group, 14 (48.3%) scored 0 points, 6 (20.7%) scored 1 point, 5 (17.2%) scored 2 points, and 4 (13.8%) scored 3 points. No significant difference was observed between the two groups (Table 6).

**Table 6:** Comparison of VFSS Findings (Non-Pneumonia Group vs. Pneumonia Group) - Pyriform Sinus Residual No significant difference was observed between the two groups. VFSS: videofluoroscopic swallowing study

Score	Non-pneumonia Group	Pneumonia Group	Test Statistic, p-value
0	20 (74.1%)	14 (48.3%)	Mann–Whitney U = 291.0; z = −1.880; p = 0.060 (two‑sided)
1	2 (7.4%)	6 (20.7%)	
2	4 (14.8%)	5 (17.2%)	
3	1 (3.7%)	4 (13.8%)	

Laryngeal Entry or Aspiration

In the non-pneumonia group, 21 (77.8%) scored 0 points, 1 (3.7%) scored 1 point, and 5 (18.5%) scored 2 points. In the pneumonia group, 12 (41.4%) scored 0 points, 7 (24.1%) scored 1 point, and 10 (34.5%) scored 2 points. The proportion of high scores was significantly higher in the pneumonia group (P = 0.015) (Table 7).

**Table 7:** Comparison of VFSS Findings (Non-Pneumonia Group vs. Pneumonia Group) - Laryngeal Intrusion/Aspiration The proportion of high scores was significantly higher in the pneumonia group (p=0.015). VFSS: videofluoroscopic swallowing study

Score	Non-pneumonia Group	Pneumonia Group	Test Statistic, p-value
0	21 (77.8%)	12 (41.4%)	Mann–Whitney U = 261.5; z = −2.424; p = 0.015 (two‑sided)
1	1 (3.7%)	7 (24.1%)	
2	5 (18.5%)	10 (34.5%)	

## Discussion

The significance of dysphagia assessment in residential care home residents

Most reports on dysphagia assessment in residential care home residents have focused on the utility of screening and evaluation tools. In one study using the Nurse Dysphagia Screening Tool in 172 care home residents, the sensitivity, specificity, positive predictive value, and negative predictive value were 72%, 92%, 50%, and 97%, respectively; sensitivity reached 100% in high‑volume homes and among later enrollees, underscoring a potential learning‑curve effect [10]. This indicates that screening tools can be effective for ruling out and detecting severe dysphagia in residential care home residents [10]. In addition, Engberg et al. reported that, among 35 residents in a specialized nursing home, the Gugging Swallowing Screen (GUSS) yielded a positive rate of 31.4%, demonstrating its contribution to identifying potential dysphagia [11]. Conversely, Estupiñán and colleagues showed that screening practices and implementation vary widely across long‑term care facilities and highlighted the need for standardized, validated protocols tailored to institutional settings [12]. These observations emphasize the limitations of directly applying methods developed in acute hospitals to long‑term care without appropriate adaptation [12]. Furthermore, scoping and Delphi‑style work in long‑term care indicates that although instrumental assessments (e.g., VFSS, FEES, mobile FEES) are theoretically valuable, their use is often constrained by logistics, staffing, equipment availability, and cost; mobile FEES may mitigate some of these barriers [13].

Our hospital addresses these challenges through collaboration with care homes. For the care homes, this approach helps prevent aspiration pneumonia and ensures the safety of feeding assistance. For the hospital, it facilitates the early initiation of swallowing rehabilitation and the timely resumption of oral intake for residents admitted from care homes, guided by pre‑pneumonia swallowing function data. Consequently, this model provides mutual benefits [5].

Although dysphagia is a well‑recognized risk factor for pneumonia in older adults, studies indicate that dysphagia alone may be insufficient to cause pneumonia in the absence of additional predisposing factors; for example, a case-control study in elderly adults showed an independent association between oropharyngeal dysphagia and community‑acquired pneumonia but also underscores that broader patient factors modulate risk [14]. Large database studies in older adults hospitalized with pneumonia further suggest that dysphagia care is underutilized despite its recognized role in aspiration pathways, implying that multiple clinical determinants beyond dysphagia shape pneumonia outcomes [15]. Prior work in ischemic stroke cohorts similarly indicates that advanced age and neurological severity are consistent predictors of stroke‑associated/aspiration pneumonia, often outweighing instrument-based findings, across diverse prediction models and multicenter datasets [16].

In contrast, our study demonstrated that, among nursing home residents, laryngeal penetration and aspiration identified on VFSSs were associated with pneumonia onset, suggesting that the presence or absence of airway invasion on instrumental assessment may serve as a critical, setting‑specific indicator for pneumonia risk stratification in this population.

Barium swallow examinations in elderly care home residents

Previous studies have reported that elderly patients with dysphagia (aged ≥ 65 years) exhibit elevated scores on the PAS, prolonged PDT and PTT, and increased pharyngeal residue [4]. In a prospective study of dysphagia secondary to frailty, PAS3, indicating pharyngeal penetration, and PAS7-8, indicating aspiration, were significantly associated with an increased risk of pneumonia during follow-up. Furthermore, the Functional Dysphagia Scale score was reported to be significantly higher in patients who developed pneumonia [17], implying a relationship between systemic frailty and impaired swallowing function. As frailty includes muscle weakness, sensory deficits, and impaired coordination, dyssynergia of the swallowing-related musculature may exacerbate pharyngeal-phase dysphagia. Furthermore, silent aspiration and pharyngeal residue are observed in elderly individuals hospitalized for aspiration pneumonia [18]. Silent aspiration, in particular, is often asymptomatic and difficult to detect through clinical examination alone, emphasizing the importance of objective assessment using VFSSs.

Moreover, patients with dysphagia, particularly older adults, may experience severe aspiration, reaching PAS8, even with small bolus volumes, likely due to reduced pharyngeal sensation and weakened protective reflexes [14]. Several studies have reported that elderly patients hospitalized for aspiration pneumonia frequently demonstrate pharyngeal-phase delay, increased pharyngeal residue, and deep laryngeal penetration progressing to aspiration (PAS3-8), indicating that pharyngeal-phase dysfunction represents the primary pathological mechanism [19].

In our study, significant differences between the non-pneumonia and pneumonia groups were observed for laryngeal penetration and aspiration, whereas individual VFSS findings such as propulsion impairment, pharyngeal residue, and delayed laryngeal elevation were not associated with pneumonia development. In addition, no significant differences were observed in overall scores for the oral and pharyngeal phases. This may reflect the characteristics of the study population, consisting of elderly care home residents requiring high levels of care (Level 3 or above), who exhibit resident-specific factors such as trunk instability and fluctuating levels of consciousness. These factors may independently contribute to pneumonia risk, separate from swallowing function. Moreover, oral care was relatively well maintained in the study facility, potentially reducing oral bacterial load. Consequently, it is plausible that some residents did not develop pneumonia despite pharyngeal clearance impairment or mild laryngeal aspiration, owing to the low bacterial burden.

In summary, although VFSS-based assessment of swallowing function provides valuable information for identifying pneumonia risk, predicting pneumonia onset in residents of elderly care facilities requires an integrated evaluation of multiple factors, including oral hygiene, physical function, cognitive and consciousness status, and dietary environment.

Limitations

This single-center retrospective observational study included a limited number of participants, and selection bias cannot be excluded. The VFSS assessment used a single 10-mL bolus, which may not reflect habitual swallowing function. The temporal relationship between VFSS findings and pneumonia onset was not controlled, and no multivariate analysis was performed, leaving potential confounding factors, such as care dependency, cognitive function, nutritional status, and oral hygiene, unaccounted for. In particular, because oral care is rigorously implemented at the study facility, these findings may not be generalizable to other settings.

## Conclusions

Among residents of elderly care facilities, laryngeal entry and aspiration on VFSSs were significantly associated with pneumonia onset. In contrast, other VFSS findings, including propulsion impairment, pharyngeal residue, and prolonged LEDT, exhibited no clear association. These findings indicate that the presence or absence of laryngeal entry and aspiration represents an important indicator for assessing pneumonia risk in this population. However, a comprehensive evaluation should consider multifactorial factors, such as care dependency, level of consciousness, and oral hygiene.
